# Deuterium Isotope Fractionation of Polycyclic Aromatic Hydrocarbons in Meteorites as an Indicator of Interstellar/Protosolar Processing History

**DOI:** 10.3390/life12091368

**Published:** 2022-09-01

**Authors:** Heather V. Graham, Jamie E. Elsila, Jason P. Dworkin, Scott A. Sandford, Jose C. Aponte

**Affiliations:** 1 Solar System Exploration Division, NASA Goddard Space Flight Center, MS-691, Greenbelt, MD 20771, USA; 2 Space Science and Astrobiology Division, NASA Ames Research Center, MS 245-6, Moffett Field, CA 94035, USA

**Keywords:** carbonaceous chondrites, hydrogen isotope ratios, PAHs, Murchison, astrobiology

## Abstract

The stable isotope composition of soluble and insoluble organic compounds in carbonaceous chondrites can be used to determine the provenance of organic molecules in space. Deuterium enrichment in meteoritic organics could be a residual signal of synthetic reactions occurring in the cold interstellar medium or an indicator of hydrothermal parent-body reactions. δD values have been measured in grains and bulk samples for a wide range of meteorites; however, these reservoirs are highly variable and may have experienced fractionation during thermal and/or aqueous alteration. Among the plethora of organic compounds in meteorites are polycyclic aromatic hydrocarbons (PAHs), which are stable and abundant in carbonaceous chondrites, and their δD ratio may preserve evidence about their formation environment as well as the influence of parent-body processes. This study tests hypotheses about the potential links between PAHs-deuteration concentrations and their formation conditions by examining the δD ratio of PAHs in three CM carbonaceous chondrites representing an aqueous alteration gradient. We use deuterium enrichments in soluble 2–5-ring PAHs as an indicator of either photon-driven deuteration due to unimolecular photodissociation in warm regions of space, gas-phase ion–molecule reactions in cold interstellar regions of space, or UV photolysis in ices. We also test hypothesized reaction pathways during parent-body processing that differ between partially and fully aromatized PAHs. New methodological approaches were developed to extract small, volatile PAHs without fractionation. Our results suggest that meteoritic PAHs could have formed through reactions in cold regions, with possible overprinting of deuterium enrichment during aqueous parent-body alteration, but the data could not rule out PAH alteration in icy mantles as well.

## 1. Introduction

### 1.1. Reduced Carbon Chemistry and the Early Evolution of the Solar System

Investigations of the origin and processing history of carbonaceous chondrites are important for understanding the formation and early evolution of the solar system. Organic compounds preserved in meteorites inform our understanding of the accretion and alteration of parent bodies. The organic carbon complement of carbonaceous chondrite can account for up to 5% of its mass and represents some of the most primitive organic synthesis products in the solar system, with origins in presolar and protosolar environments and relatively undifferentiated by comparison with other meteorites [1]. The organic inventory of carbonaceous chondrites includes a vast array of thousands of compounds ranging from small volatile molecules to highly branched insoluble macromolecules and includes polycyclic aromatic hydrocarbons (PAHs) in both the solvent-soluble and insoluble portions of the organic matter [2,3,4,5].

PAHs are among the most ubiquitous extraterrestrial organic molecules and may represent up to 50% of the carbon in space [6,7,8]. PAHs are fused aromatic hydrocarbon rings believed to be produced in the outflow of aging stars [9,10]. PAHs are formed either by “top-down” cleavage reactions or “bottom-up” formation from precursors [11], such as UV radiation of soot particles [12] or dust particles [13] for the former and gas-phase ion–molecule reactions on grain surfaces as an example of the latter [14]. These compounds have been detected in protoplanetary and protostellar nebulae [15], dense molecular clouds, in ices and grains of the diffuse interstellar medium [16], on interplanetary dust particles [17], and in the comae of certain comets [18]. Like all presolar species, PAHs and related aromatic compounds are incorporated into asteroids and other parent bodies, where they remain among the most common soluble organic compounds in carbonaceous chondrites and are preserved in the mineral matrix [19,20,21]. Soluble PAHs can be fully or partially aromatic and can accommodate alkyl group and heteroatom substitution [22,23,24,25], while the insoluble macromolecular organic matter in meteorites is made up of PAHs and other aromatic compounds bonded by highly branched aliphatic chains [2]. This process of accretion and alteration in the parent body can affect the chemical and structural makeup of organic constituents, but the stability of PAHs allows them to traverse this immense time and turmoil with only minor alteration [26]. PAHs can serve as vestiges of the chemical evolution of our early solar system, from stellar outflows to planetary formation [9], and the record of this history is locked in meteorites.

While PAHs can be used to understand the early solar nebulae, the history and residence times of molecules in different interstellar environments are not well known. PAHs and other aromatic molecules represent a known link between the interstellar medium and solar system samples and may potentially serve as probes of the chemical processes associated with interstellar deuterium fractionation [27]. Although the carbon isotope ratio (^13^C/^12^C) of PAHs is likely to be established during their formation in stellar outflows, the deuterium isotope ratio (D/H) of PAHs will reflect their processing history. The D/H composition can be affected by the environment the molecules are in as the peripheral hydrogen atoms exchange or react [9]. The incorporation of deuterium into PAH molecules can then help us understand the relative importance of various interstellar and protosolar reaction environments and parent-body alteration processes on the organic complement of meteorites [28]. Specific predictions have been made about the signatures expected from various chemical reactions and conditions [25,28,29,30,31]. Carbon isotope ratios have been used to demonstrate the extraterrestrial origins of other organic compounds such as amino acids and carboxylic acids in meteorites [31,32,33,34,35]. Similarly, this study attempts to reconstruct the conditions of these interstellar/protosolar environments by querying the deuterium enrichment patterns recorded in meteoritic PAHs.

Prior analyses of PAHs in meteorites have reported qualitative assessments of PAH content in a variety of petrologic chondrite types [36] and bulk δD isotope measurements of aromatic fractions [37], but only two studies report the compound-specific δD composition of meteoritic PAHs for unaltered CM class chondrites [38,39]. This study expands on these data by performing compound-specific deuterium isotope analysis of a targeted suite of extracted PAHs across a range of meteoritic petrologies to test proposed enrichment mechanisms and further develop our understanding of the origins and alteration history of PAHs in early solar system chemistry.

### 1.2. The Role of Deuterium in Understanding the Origin of PAHs

Given their longevity, PAHs record the hydrocarbon chemistry of the early organic evolution of our solar system. The extent of deuterium enrichment in presolar species such as PAHs may be an indicator of these early interstellar reaction environments. Previous work predicted a number of potential reaction pathways that could lead to different expressions of deuterium enrichment in meteoritic PAHs [28,29,38]. These reactions occur in several different astrophysical environments, including (1) interstellar environments such as photodissociation regions in which PAHs exist in the gas phase, (2) the gas phase of cold dense molecular clouds, (3) environments where icy grain mantles are exposed to radiation, and (4) meteorite parent bodies (Table 1). This study compares these predictions with experimental measurements of the D/H ratio of PAHs extracted from meteorites to see which proposed pathways are consistent with the meteoritic values.

Enrichment patterns in PAHs can indicate the interstellar and protostellar environments that have been important in modification of the molecules and help provide context for the provenance of these molecules. In most cases, deuterium enrichment reactions occur because of the zero-point energy differences between a D and H atom on PAHs, which lead to C-D bonds that are slightly stronger than C-H bonds, thus causing preferential breaking of the bonds with the lighter element over the bond with the heavier element [9,41,42]. Two models of PAH alteration suggest deuterium enrichments can occur in both warm and cold regions of space [28,29]. Deuterium enrichments can occur on gas-phase PAHs over a wide range of temperatures if they are exposed to ultraviolet radiation that can drive unimolecular photodissociation. A cold origin for the D-enrichment of PAHs can occur via gas-phase ion–molecule reactions in cold dense molecular clouds or UV photolysis of PAHs in D-enriched ices. Patterns of PAH deuterium enrichment are expected to differ in these regions of space and each of these reactions should produce a distinctive signature δD composition when compared with certain structural PAH properties (Table 1).

In this study, we report the identity and compound-specific δD composition of 2–5-ring unfunctionalized PAHs in three CM carbonaceous chondrites with similar thermal histories but differing amounts of aqueous alteration. We compare the δD composition trends across molecular weight, structural types, and alteration histories to test the four predicted reactions that vary in space, time, and temperature. These mechanisms and locations include possible gas-phase unimolecular dissociation reactions, gas-phase ion–molecule reactions in dense interstellar clouds and the cold regions of protosolar disks, irradiation of PAHs in D-enriched ices of those dense clouds and the cold regions of protosolar disks, and aqueous alteration processes in the body of an asteroid.

## 2. Methods

*Sample Selection and Provenance:* Three CM carbonaceous chondrites were selected for this study: Allan Hills (ALH 83100, Specific 294, parent 22; 5.1 g), Lonewolf Nunataks (LON 94101, Specific 97, Parent 5; 5.2 g), and Murchison (5.2 g). The first two were provided by the Antarctic Meteorite Collection curated by the Astromaterials Acquisition and Curation Office at NASA Johnson Space Center. Murchison was from the Chicago Field Museum, via the Clifford N. Matthews research group. These three samples were selected based on the desired comparison with previous qualitative work and constrained petrologic range, as well as an ample available sample mass. All three samples represent the same petrographic type but with a gradient of aqueous alteration, with ALH 83100 representing the most aqueously altered of the three with an alteration of 1.1 on the Alexander et al. scale [43] and 2.1 on the Rubin et al. scale [44], Murchison of intermediate alteration (1.6 or 2.5 on the two respective scales), and LON 94101 the least aqueously altered (1.8 or 2.6 on the two respective scales).

*Sample Preparation and Extraction:* All glassware and ceramic materials used for sample preparation were wrapped in aluminum foil and baked in air at 500 °C for 8 h to remove organic contamination. Each ~5 g sample was split into two portions of approximately 2 and 3 g and powdered in a ceramic mortar and pestle then transferred to a clean glass vial with a PTFE cap (that had been cleaned by the following solvent extraction method) and sonicated (Branson 3800 Ultrasonic Cleaner) at room temperature for thirty minutes with three 2 mL aliquots of methanol (VWR Omnisolv, 99.9% purity) followed by two 2 mL aliquots of dichloromethane (DCM) (VWR Omnisolv, 99.9% purity). Methanol and DCM extracts were combined and treated with elemental Cu beads (Costech, #B1015M) to remove extracted sulfur-bearing species that interfere with compound-specific isotopic analyses. Prior to use, Cu beads were cleaned by three washes of 1.0 M HCl (Sigma Aldrich, ACS grade 37%) to remove the oxidized surface and then washed three times in both extraction solvents (methanol and DCM). PAH standards included the EPA Method 8310 16-component PAH Mixture (610 PAH calibration mix Restek #31455) and biphenyl (TCI America, >99.5% purity).

*Sample purification:* Extracts were concentrated using a custom-built spinning-band distillation apparatus (Ace Glass) that gently evaporates volatile low-molecular-weight components in an organic solvent while returning higher molecular weight less-volatile species to a heated vessel (further details in the section below). A high-precision liquid chromatography (HPLC) purification step was used to separate three- and four-ring species into fractions using a Restek Pinnacle II PAH column (3.0 × 150 mm, 4 µm particle size, 110 Å pore size) and gradient of A 1:1 methanol–water and B acetonitrile (VWR Omnisolv, >99.9% purity). The column flow rate was 0.5 mL/min starting at 50% for 2 min, a linear ramp to 90% B over 2.5 min, and a linear ramp to 100% B for another 2.5 min before holding for 10 min. Water was from a Millipore Integral 10, 18.2 MΩ·cm, <3 ppb TOC, and acetonitrile was Fisher Optima grade. These fractions were again concentrated by spinning-band distillation. Residual water was removed by adding sodium sulfate (Alfa Aesar, >99% purity, cleaned by 2:1 DCM–methanol extraction solvent prior to use) to the distilled fraction and allowing it to sit overnight.

*D/H Isotope Analysis:* Compound-specific isotope analyses were performed using a hybrid gas chromatography and isotope-ratio mass spectrometry (GC-MS/IRMS) instrument consisting of a Thermo Scientific Trace GC connected in parallel to a DSQ II quadrupole mass spectrometer and a MAT 253 isotope-ratio mass spectrometer with a Thermo Scientific GC/TC high-temperature pyrolysis furnace held at 1400 °C [45]. For simultaneous analysis of isotope ratio as well as mass spectrometry, 90% of the injected amount was directed to the combustion interface with the IRMS, while the remaining 10% was directed to the DSQ for compound identification [35]. The daily average H_3_^+^ factor was recorded throughout the analytical time frame and was maintained below 10.5 ppm/nA for all analyses. The H_3_^+^ factor indicates the contribution of H_3_^+^ species formed by reactions in the ion source with increasing gas pressure and can affect the accuracy of the measured δD value. Isotopic composition was determined relative to a custom reference gas calibrated to an international standard (Oztech Corporation, δD_SMOW_ = +100‰) as well as by regular coinjections of a calibration mix (Restek 610 PAH calibration Mix B) and a biphenyl standard (Arndt Schimmelmann, Indiana University, Bloomington, IN, USA, δD_SMOW_ = −41.2‰). Compound identification was based on comparison with standards, elution time, and mass spectra, although identification of the largest six-ring species is tentative given the many isomers of these larger PAHs. Systematic instrument errors were determined by repeated analyses of internal standards and corrected for sample size. δD values were reported relative to Standard Mean Ocean Water (SMOW). Accuracy and instrument precision of measurements was found to be ±11.0‰ (*n* = 36) and ±70.0‰ (*n* = 36), respectively, and was determined by comparison to the measured and true D/H value of the biphenyl standard.

Bulk isotope analyses and weight percent total hydrogen values were performed on a high-temperature conversion elemental analyzer (Costech TC/EA) coupled to the isotope-ratio mass spectrometer housed in the Schimmelmann labs at Indiana University. Samples were pyrolyzed at 1350 °C before chromatographic separation of H_2_ gas in a packed column held at 85 °C. Isotopic composition was determined by repeated analyses of the international polyethylene foil standard (IAEA PEF, δD_SMOW_ = −97.15‰) and additional alkane and polyethylene powder standards (from Arndt Schimmelmann, Indiana University; *n*-C_36_ δD_SMOW_ = −259.0‰ and PE powder δD_SMOW_ = −75.9‰). Samples were weighed into baked silver capsules (#041066, Costech). To remove adsorbed water, samples were pumped under vacuum for 24 h before analysis. Accuracy and instrument precision of measurements was found to be ±2.1‰ (*n* = 12) and 5.2‰ (*n* = 12), respectively, and was determined by comparison to the measured and true D/H value of the three standards. Values are reported as the average of triplicate measurements.

### Prevention of Volatile Loss and Limitation of Isotope Fractionation

Sample handling procedures were designed to minimize the risk and impact of isotopic fractionation of analytes and to prevent the loss of the smallest (two-ring) PAH species. The focus on preserving these volatile species was necessary to test the proposed enrichment mechanisms in Sandford et al. [29], which required analysis of the widest possible size range of soluble PAHs. Prior to extraction and analysis of the meteorite samples, a series of extraction and solvent reduction tests were performed on the NIST Standard Reference Material 1941B (Organics in Marine Sediment) specifically designed for PAH method development. Further, the most promising solvent reduction steps were also tested with the Restek 610 PAHs standard calibration mix to quantify any isotopic fractionation arising from the method.

Additional extraction steps (exceeding those listed in the methods above) were tested. These included extra sonication steps in either hexanes or acetonitrile or a combination of DCM–MeOH. These steps did not significantly affect extraction efficiency. Similarly, it was found that a heated sonication step decreased the yields of the lightest-molecular-weight species (naphthalene, biphenyl, acenaphthene, and acenaphthylene) by 15–40%.

Five solvent reduction methods (blowing nitrogen dry down, chilled centrifugal evaporator (Labconco Centrivap), lyophilization (Labconco FreeZone), combined spiral plug nitrogen flow plus vacuum (Biochromato Smart Evaporator), and a custom spinning-band distillation apparatus) were evaluated for concentration of the combined extracts to assess the loss of low-molecular-weight PAHs and isotope fractionation. Centrifugal evaporation, lyophilization, and spiral plug dry down all resulted in near-complete loss of the lightest 2-ring PAHs. Blowing nitrogen dry down was attempted both at room temperature and in a chilled reservoir, with similar results showing relatively little loss of the lightest-molecular-weight species; however, an accidental complete or near-complete dry down of an extract resulted in large losses of 2-, 3- and 4-ring PAHs.

**Figure 1:**
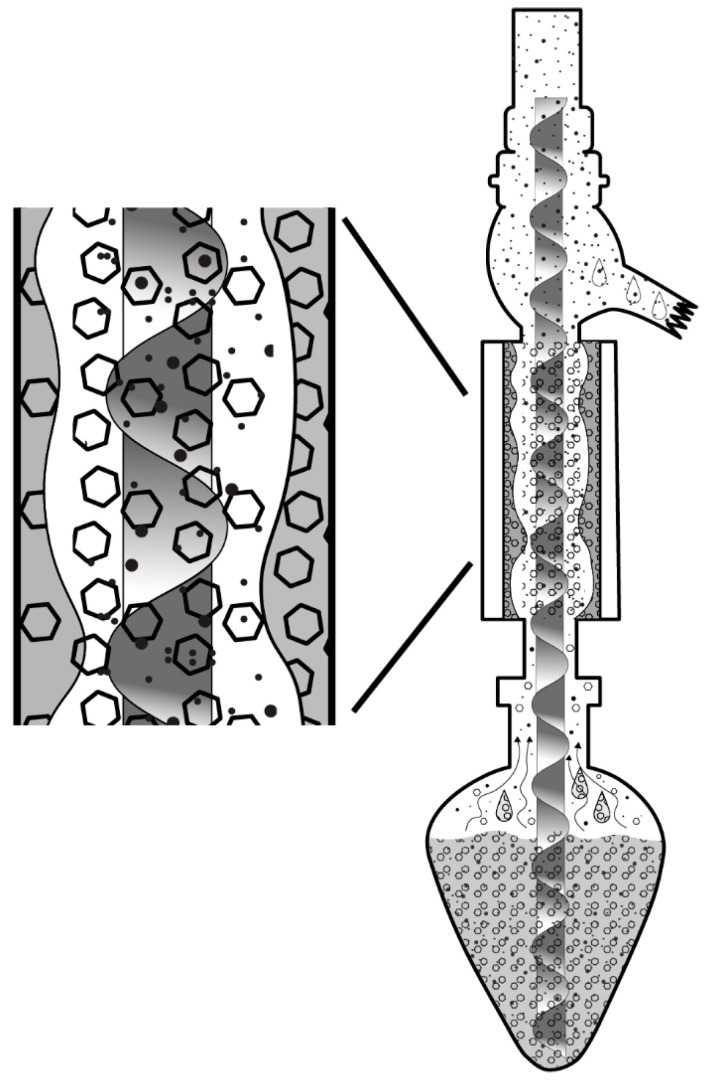
Slow evaporation by spinning band removed DCM and most of the methanol (large and small circles) with little loss or isotopic fractionation of PAHs (hexagons). As the vessel is heated, solvent evaporates through the column, but heavier dissolved species will recondense on the Teflon spinning screw and return to the reservoir, slowly accumulating as solvent is removed.

By far, the method with the least potential for low-molecular-weight species loss or fractionation was the spinning-band distillation column (Figure 1). The efficacy of this method has been previously demonstrated in a study that compared PAH losses for six solvent reduction methods [46]. The spinning-band method of solvent reduction has the highest recovery of low-molecular-weight and low-boiling-point PAHs, such as naphthalene. Using a custom-made apparatus, extracts were concentrated with less than 5% loss of any of the tested 2–5-ring PAH standards. The spinning-band distillation apparatus concentrates extract in a reservoir flask that is immersed in a hot water bath on a stirring hot plate. The flask is connected to an insulated distillation column with a length of rotating threaded PTFE screw (the “band”). As the solvent vapor travels through the distillation column, the rotation of this band returns higher-molecular-weight species to the extract in the reservoir. The spinning is enabled by a metal knob at the bottom end of the PTFE screw and operated by the stirring function of the hot plate. The reservoir flask in the water bath is kept just below the boiling point of the methanol–DCM solvent mix (~50 °C) to prevent loss of low-molecular-weight PAHs.

## 3. Results

**Figure 2:**
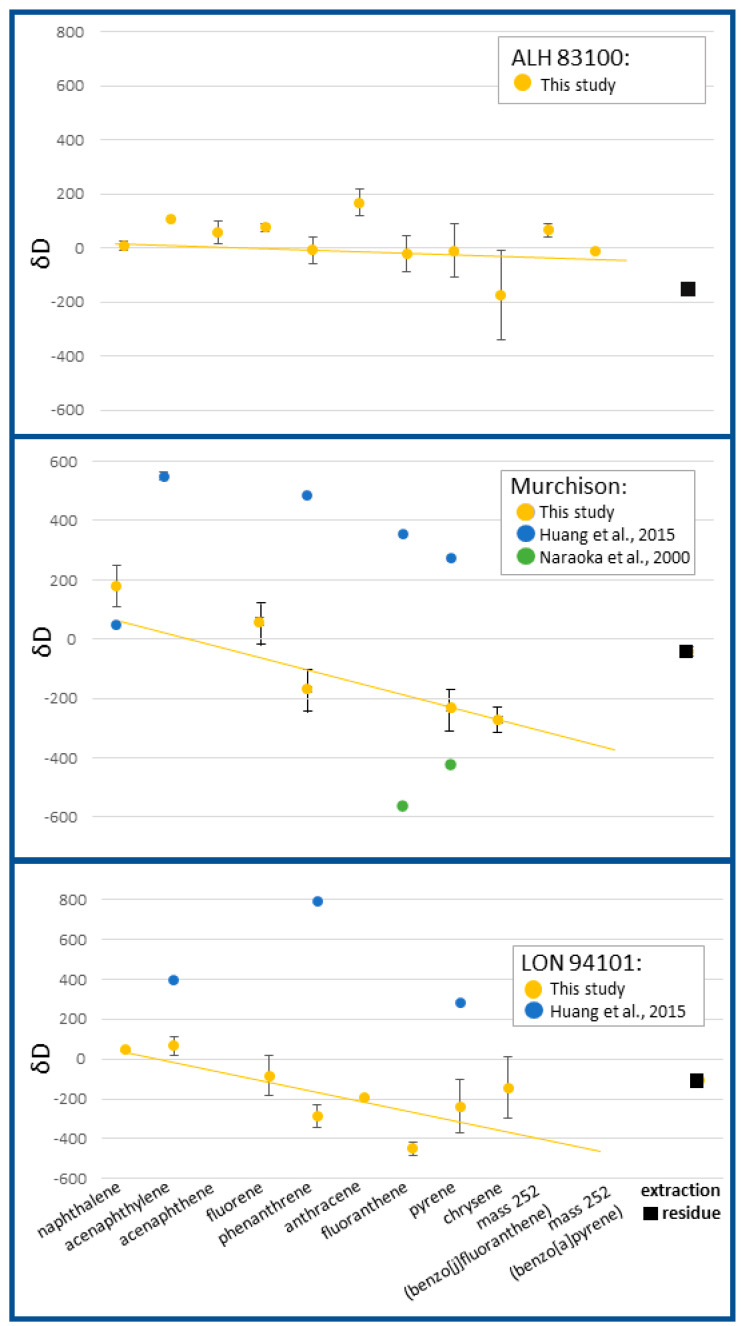
δD measurements for the 11 identified PAH species outlined in Table 2. The *x*-axis is arranged in order of increasing molecular mass for soluble species, with the δD value of the extraction residues presented last. Additional D/H values from Huang et al., 2015 and Naraoka et al., 2000 [39,40] are included for comparison. The meteorites are arranged vertically from most altered (ALH 83001) to least altered (LON 94101). Trendlines are calculated for PAHs by the average δD of ring numbers of the soluble species shown.

The results of the deuterium stable isotope ratios for PAHs from ALH 83100, Murchison, and LON 94101 are presented in Table 2. Error was determined by repeated analysis of internal standards. The δD values range from +180‰ to −450‰. Within individual meteorites, the isotopic range was as little as ~340‰ for all PAH species identified (ALH 83100) and as much as ~515% (LON 94101). The chondrite with the widest δD range, (LON 94101), was the most altered of the three chondrites studied. In general, the higher the molecular weight, the higher the ring number of PAHs in the two more altered samples (Murchison and LON 94101) are increasingly more depleted in D (Figure 2).

Bulk D/H measurements of the post-extraction residues (Table 3) represent a bulk value for recalcitrant or mineral-bound organic matter (Figure 2). This value reflects both aromatic and aliphatic moieties; while there could be a diverse range of molecular-weight molecules, the residue also contains the insoluble aromatic macromolecules with greater than 40 carbon atoms.

## 4. Discussion

### 4.1. Generally Light Isotopic Values for Isolated PAHs

δD isotope values for PAHs extracted for this study differed from previously reported values (see Murchison and LON 94101 values in Table 3); however, these previous studies measured δD on materials that had been processed for other analyses. Although there is not an abundance of data from those studies to compare extraction efficiencies, it is possible that these sample preparation differences affected the measured species. For example, Huang et al. [39] measured δD in PAHs from a heated solvent extraction of meteorite residues that had previously been subjected to a hot water extraction. Isotopically lighter species were likely lost during the warmer extraction step, while lower molecular weight and partially aromatic species that are slightly water-soluble could have been lost in the initial hot water extraction protocol (for example, the solubility of naphthalene in water at 25 °C is 0.25 mM). In contrast, the focused extraction and solvent reduction steps used in our current work differed significantly from these previous studies and were optimized to minimize loss of small-molecular-weight species. Thus, while the relative isotopic trend remains the same, the absolute enrichment of these extracts could differ, explaining the lighter δD values we measured compared to Huang and coworkers [39]. Similarly, Krishnamurthy et al. [37] observed an enriched δD value for a bulk aromatic fraction (see Table 3) for a meteorite residue that had previously been extracted in water and methanol.

Values measured in our current study are also more enriched than those presented by Naraoka et al. [38]; however, that study used a much smaller portion of meteorite (~1/8th the sample mass used in this study) that was also extracted in a 3:1 benzene–methanol mixture and purified by short-column silica gel chromatography [47]. These differences in sample size, preparation, and extraction may have resulted in enhanced dissolution of deuterium-depleted species. It is possible the observed decreased solubility of deuterated species in a nonpolar solvent could have resulted in less of the D-enriched PAHs in the fraction analyzed, a process observed in deuterated standards [53]. Based on these comparisons for soluble aromatic molecules, this study shows that extraction methods can have a substantial effect on isotopic expression and indicates that soluble species are generally isotopically light when compared with insoluble organic matter.

### 4.2. PAH Origin Estimations Based on D/H Trends with Ring Size

**Scheme 1:**
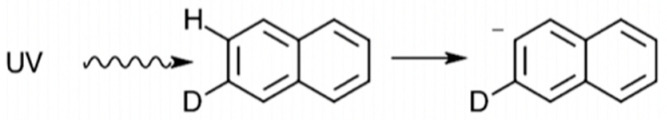
Unimolecular photodissociation.

The results from our analyses suggest that PAHs in CM2 chondrites are systematically depleted in D with increasing size of the molecule (Figure 2). However, this depletion trend was less pronounced in the most aqueously altered sample, ALH 83100. Using the interpretive framework based on Sandford et al. [29], a depletion with PAH size pattern is consistent with enrichment of gas-phase PAHs by unimolecular photodissociation (Figure 2), a process that can occur in some interstellar and protosolar environments. Unimolecular photodissociation is a photon-driven reaction that preferentially enriches smaller PAHs and is unlikely to affect other species. Given their abundant complement of p electrons and C=C bonds, PAHs are much more stable than other molecules against photolytic disruption. PAHs can absorb most of the energy of an interstellar UV photon with only the occasional breaking of a peripheral C-H bond (Table 1) [28,54]. This site can be replaced by either a D or H, but gradually, over time, these sites will be dominated by D due to the greater strength of the C-D bond, resulting in D-enrichment (Scheme 1) [9,55].

This effect is likely to be most pronounced in PAHs having fewer than ~40 C atoms, since larger molecules have greater numbers of vibrational modes that can accommodate UV photon bombardment and can better retain their original peripheral hydrogen atoms [55] re-emitting the added energy as a cascade of infrared photons, rather than breaking a peripheral carbon-hydrogen bond [56,57]. In our study, very large aromatic molecules (>40 C atoms) would be retained in the nonsoluble bulk residue. These large molecules would not be affected by unimolecular photodissociation and are generally expected to match the bulk D/H value of the original PAH. Our measured δD values for the bulk nonsoluble residue (which would include these large PAHs as well as other species and mineral phases) for each of the three chondrites were slightly enriched (30–50‰) when compared with previously reported bulk δD measurements, reflecting the loss of the more depleted soluble aromatic species.

**Scheme 2:**
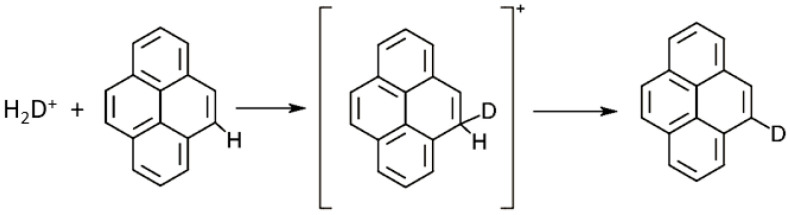
Gas-phase ion–molecule reactions.

**Scheme 3:**
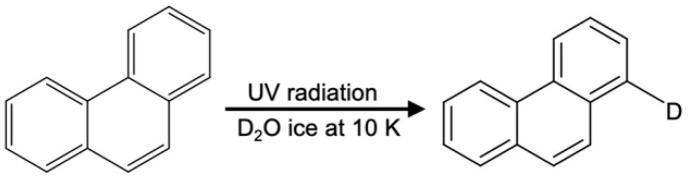
UV photolysis in ice.

In contrast to unimolecular photodissociation, gas-phase ion–molecule reactions (Scheme 2) and ice photolysis reactions (Scheme 3) require low-temperature conditions and are thus more likely in pre-accretionary environments. These reactions are widely accepted as a deuterium enrichment mechanism for many species in the interstellar medium [41,58]. The gas-phase ion–molecule enrichment mechanism is a two-step process where, first, a PAH molecule in dense interstellar clouds will become charged by reacting with a H_2_D^+^ ion (Scheme 2). An electron then neutralizes the charge while eliminating the extra D or H atom. As discussed before, the C-D bond is stronger than C-H, resulting in a gradual accumulation of deuterium at affected bonding sites.

Similarly, the process of enrichment by photolysis in ices is the energetic processing of molecules in dense clouds of interstellar ices by cosmic rays that include the diffuse UV field in the interstellar medium as well as UV photons produced by stars forming within the cloud [59]. As deuterium-rich ices created by gas–grain reactions [60] are irradiated and warmed, complex chemistry occurs within the molecules condensed in these ices leading to bond breakage, radical and ion formation, and possible molecule destruction [28]. These molecules could include PAHs previously D-enriched by other reactions that have concentrated in the ice mantles where further D-enrichment will occur by D-H exchange reactions at nonlabile locations during the ice phase irradiation process [28,61] (Scheme 3).

While gas-phase ion–molecule enrichment reactions do preferentially occur on PAHs with higher molecular weights, over time, the end result of many repeated reactions would likely not exhibit any enrichment trend with molecule size, ring number, condensation, or functional group substitution [29]. By contrast, ice photolysis reactions are size independent, where every molecule is equally likely to interact with D-enriched ice. While these two mechanisms could yield the same PAH enrichment results, ice photolysis processes would also produce distinctive deuterated oxidized and reduced species (heterocyclic aromatic compounds and H_n_-PAHs) [28].

The less variable D/H values observed in the more aqueously altered ALH 83100 (CM1/2) chondrite compared to the CM2 chondrites (Figure 2) could be consistent with enrichment by gas-phase ion–molecule reactions or with photo-induced exchange in D-enriched ices. However, given that the CM chondrites are often assumed to all arise from the same parent body [62] or same type of parent body [63], this interpretation would require ALH 83100 to represent a significantly different portion of the parent body. Previous carbon and nitrogen analyses of CM1 and CM1/2 meteorites have found higher variability between fragments that may result from the variation in matrix contents vs. chondrules [43,64]. However, the reported δD values of materials in the matrix vs. chondrules is not similar in CM chondrites [65], either for specific organics or bulk components. Alternatively, the higher degree of aqueous alteration could also affect the isotopic values of PAHs in unique aqueous alteration conditions [66]. Thus, the differences observed between the CM2 and CM1/2 chondrite in our study may be related to increased aqueous alteration on the parent body.

### 4.3. Parent-Body Alteration and PAH Deuterium Enrichment

Parent-body processing can link meteoritic aqueous alteration and petrology [25,29,38] but the extent to which these reactions may affect the isotopic expression and molecular distribution in PAHs is unknown. This makes it difficult to deconvolve enrichment effects by low-temperature reactions versus those that may arise during parent-body processes, such as aqueous alteration. The molecular distribution and δD composition of meteoritic PAHs can help us understand the relative importance of various parent-body alteration processes.

**Figure 3:**
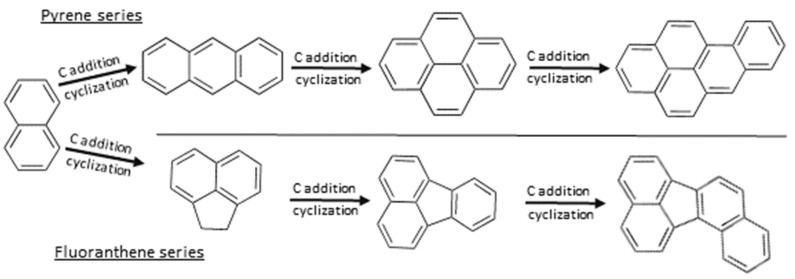
Two possible pathways for forming larger peri-condensed PAHs from smaller homologs, suggesting that isotope distribution is kinetically controlled, producing fractionation during carbon formation and cyclization that adds rings. Concept and drawing after Naraoka et al. 2000 [40].

An example of a parent-body alteration process that could affect PAH enrichment is aromatic cyclization reaction pathways that build larger PAH molecules through ring addition (Figure 3). These kinetic reactions in a parent body have been proposed as the mechanism behind observed distinct patterns of δ^13^C expression in PAHs extracted from primitive meteorites [40] and could result in distinctive δD depletion trends for molecules of nearly the same molecular weight [38]. These trends relate to ring number as well as ring condensation (H/C) with a 10‰ difference in δ^13^C between the fully aromatic species with exclusively 6-membered rings (pyrene family) and partially or fully aromatic species that contain both 5- and 6-membered rings (fluoranthene family) (see Table 2). The relationship between H/C ratio and δ^13^C suggests distinct cyclization pathways by which larger rings are formed from smaller, less ^13^C-enriched rings [67], perhaps during parent-body processing and alteration [29]. Similar kinetic reactions have been proposed to explain δ^13^C trends in meteoritic monocarboxylic acids [68].

**Figure 4:**
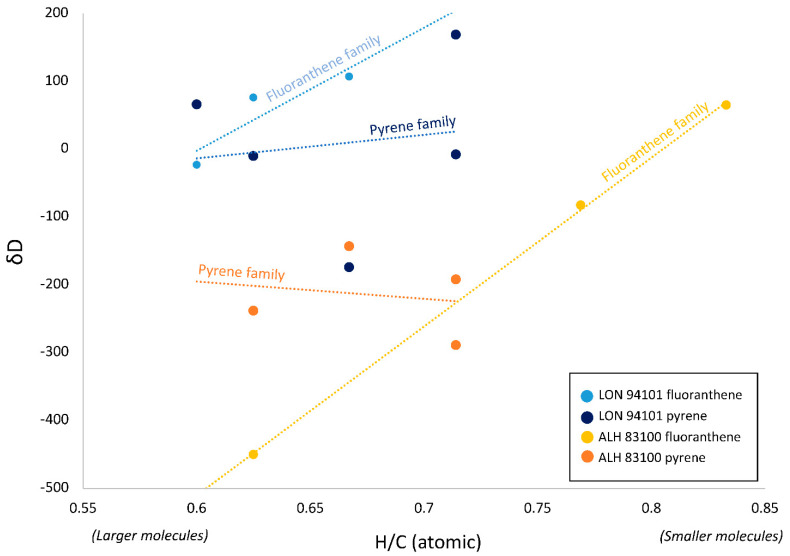
Chondrite PAH δD composition trends with condensation and approximate molecule size show D-depletion with increasing molecular mass. Distinctive trends with D content suggest independent PAH formation (cyclization) pathways where partially aromatic species are preferentially enriched as additional rings are added. As larger homologs are formed there is a loss of D relative to carbon and this is more evident in the nonalternant fluoranthene family species.

Ring-building by kinetic cyclization reactions could result in patterns of deuterium depletion since the zero-point energy difference between C-D and C-H bonds requires more energy to break D-H bonds, leading to an overall slower rate of cyclization that involves deuterated rings. This would lead to a δD signature of depletion with increasing molecular weight. Results from our study did show this characteristic depletion for the partially aromatic species, but less so for the fully aromatic species (Figure 4). The partially aromatic family compounds range from +100‰ to −450‰, while the fully aromatic species range from +100‰ to −250‰. While there was an offset in the overall isotope composition, there was no greater D-depletion for the more aqueously altered meteorite, suggesting that kinetic cyclization reactions are not necessarily related to aqueous alteration but perhaps some other parent-body process.

### 4.4. Comparisons with Previous Data Indicate D/H Depletion in Soluble Species

While it is difficult to draw conclusions with data from only three chondrites, we can contextualize these data within the greater array of δD data that has been collected on bulk meteoritic material, insoluble organic matter (kerogen) residues after acid treatment of a chondrite, bulk extractions of only aromatic fractions, and the rare examples of compound-specific PAH δD measurements. These data have been summarized for CM2, CM1/2, and CI1 chondrites, including the three examined in this study chondrite (Table 3). This comparison allows us to make broader statements about effects of aqueous alteration and also to understand the isotopic expression of individual compounds within the greater context of the organic complement of a chondrite.

It might be expected that the deuterium enrichment in bulk extraterrestrial materials would be dominated by the great quantities (79 to 99% of organic carbon by weight) of insoluble organic matter found in carbonaceous meteorites [3]. However, although much of the carbon in a CM meteorite is found in the IOM only ~30% of this carbon is hydrogenated [69]. This is evident in the difference between the enriched δD values of the bulk compared with the much more isotopically D-depleted soluble organics. Using ALH 83100 as an example, the bulk rock (including all organics and minerals) has a D/H value of −201‰ ± 0.5‰, where the IOM is enriched at +723‰ ± 11‰ but the individual extracted compounds (the bulk of the organic hydrogen) range from +169‰ to −174‰. These more intermediate compound-specific measurements help drive the bulk δD expression to a more depleted value. By comparing the bulk D/H value with the insoluble organics it is evident that the soluble (and possibly hydrated mineral) fractions of chondrites are greatly D-depleted. While some of this isotope depletion may be incompletely desorbed terrestrial water [43], data collected in this, and other, studies confirm that the soluble species are comparatively D-depleted (see Table 3). Further, both the compound-specific isotope measurements of isolated IOM components [52] and the pyrolysis of IOM [69,70,71,72] indicate that there is a D/H enrichment dependent on C-H bond type, with aromatic bonded components the most depleted, followed by aliphatic and then benzylic components. Laboratory simulations using UV photons to irradiate an IOM-analog polymer also produced these same D-enrichment patterns with functional groups and replicated the ratios observed in the Orgueil chondrite [73]. Thus, aromatization in meteoritic organic compounds is, in general, associated with isotopically lighter hydrogen values.

There is one example in this data compilation of a bulk aromatic extraction [37] that does not follow this prediction for depleted aromatics. This value was measured on a meteorite residue that had previously been extracted in both water and methanol and, similar to the values for Huang et al. [40], this process of extraction may have skewed the values for the remaining aromatic compounds towards being more D-enriched. Hot-water extraction may solubilize isotopically lighter PAHs which were not measured during the analysis of more polar species. With this in mind, there does seem to be more evidence of δD-enrichment in aromatic species in the more aqueously altered chondrites included here. The vast difference in solubility with PAH ring number and molecular weight could also suggest that the more soluble PAH species (such as naphthalene vs pyrene or fluoranthene) would be subject to increased aqueous alteration or could also serve as a mechanism for increased transport, sometimes referred to as geochromatography, within the parent body [74].

While this study focused on CM chondrites, previous studies measuring the δD of CI chondrites could build expectations for returned samples Ryugu and Bennu. For example, bulk rock δD for the Orgueil CI1 chondrite (Table 3) was D-depleted, while the IOM and isolated aromatic compounds were D-enriched. This could indicate that Orgueil (and other CI1 chondrites and perhaps Ryugu [30]) occupy different typical isotopic compositions than CM chondrites which could relate to parent-body differences [75], as well as Orgeuil’s extensive aqueous processing history of CI1 [76]. Likewise, the enriched values for the two isolated PAHs from Orgueil could be a continuation of the trend detected in the three samples examined in this study that found general increased D-enrichment with aqueous alteration regardless of trend with molecule size. This could also suggest that any effects of deuterium incorporation recorded during PAH formation in the different stellar environments discussed could be overprinted by later parent-body processing, as seen in the more altered chondrites. In general, the comparisons summarized in Table 3 indicate that individual PAH δD values are decoupled from the bulk and IOM δD for the CM chondrites.

## 5. Conclusions

In this study, we report the identity and compound-specific δD composition of 2–5-ring PAHs in three CM carbonaceous chondrites with similar thermal histories but differing amounts of aqueous alteration. The data presented do not match with the previously collected values in Huang et al. [39] or Naroaka et al. [38], but this was not unexpected given the methodological differences in sample preparation since, unlike in this study, the earlier work did not prioritize the preservation of the smallest PAHs thus their results should be viewed with caution. The reported δD values all fall within ranges similar to those in general observations for bulk materials. While there are many mechanisms that affect the deuterium incorporation into meteoritic PAHs, our data from the most aqueously altered CM1/2 meteorite we examined, ALH 83100, shows a relatively flat amount of D-enrichment with increasing ring size and molecular mass. This is consistent with enrichment processes involving gas-phase ion–molecule reactions and/or photolysis-driven exchange in the D-enriched ices, both processes constrained to occur in very cold environments. In contrast, the less aqueously altered CM chondrites Murchison and LON 94101 show a trend of decreasing δD with increasing ring size and molecular mass, consistent with D-enrichment via photon-induced unimolecular dissociation. Taken in aggregate, there was enrichment in all PAHs with aqueous alteration across this selection of chondrites. This connection to aqueous alteration is also supported by the different cyclization pathways evident in the two populations of pyrene and fluoranthene family compounds.

Given the complexity of the many possible processes, these data can only suggest some of the possible mechanisms that have affected deuteration in PAHs. However, our study provides support for previously suggested formation and alteration mechanisms for these meteoritic compounds. Additional PAH δD datasets that include a greater span of alteration conditions will be necessary to deconvolve these many effects. For example, compound-specific PAH δD data from CI or CR chondrites could help indicate the influence of aqueous alteration and thermal metamorphism. Additional data collected from CI chondrites could help build hypotheses for PAHs in the material from Ryugu returned from the Hayabusa2 mission [30] as well as material from Bennu, soon to be returned by the OSIRIS-REx mission [77]. Aqueous alteration on Bennu appears to be heterogenous and thus could potentially offer a way to test the hypothesized PAH D-enrichment alteration with material known to arise from the same parent body. Trends in D-depletion in the less altered samples returned could inform our understanding of the PAH mechanism and help deconvolve the effects of the warm environment reactions such as unimolecular photodissociation from those originating in cold regions, such as gas-phase ion–molecule reactions and UV ice photolysis.

## Figures and Tables

**Table 1:** Patterns of deuterium enrichments in PAHs can serve as signatures for specific reactions and environments where PAHs were formed or processed.

Reaction/Environment	Mechanism/Location	Deuteration Pattern
Unimolecular photodissociation (warm and cold environments, UV photon-driven)	Gas-phase PAHs in interstellar medium or dense interstellar molecular clouds [29]	Smaller PAHs more enriched, no effect on >40 carbons
Gas-phase ion–molecule (cold environments)	Gas-phase PAHs in cold dense interstellar molecular clouds or cold regions of protosolar disks [28,29]	No enrichment trend with molecule size or ring number
Photo enrichment of PAHs in D-enriched ices (cold environments, UV photon-driven)	PAHs condensed in ices in dense interstellar molecular clouds or cold regions of protosolar disks [29]	No enrichment trend with molecule size or ring number (enrichment correlates with functionalization)
Cyclization pathways (kinetic reactions in parent body)	Larger PAH formation by ring addition in chondrite parent body [40]	Differs between partially and fully aromatized PAHs

**Table 2:** δD values * for PAHs from the three chondrites analyzed in this study. Species are grouped as either members of the fully aromatic or “alternant” pyrene family molecules or as members of the “nonalternant” fluoranthene family of molecules that include five-membered rings and can be partially aromatic. The table also presents molecular formula, molecular weight, and the ratio of hydrogen to carbon atoms for each species.

Structure and Name	Formula Mass H/C	ALH 83100 δD (SMOW)	Murchison δD (SMOW)	LON 94101 δD (SMOW)
Pyrene Family: Six-Membered Fully Aromatic (Alternant) PAHs				
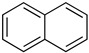 Naphthalene	C_10_H_8_ 128 Da 0.8	7‰ ± 18‰	180‰ ± 70‰	46‰ ± 6‰
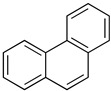 Phenanthrene	C_14_H_10_ 178 Da 0.714	−8‰ ± 50‰	−170‰ ± 8‰	−289‰ ± 57‰
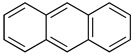 Anthracene	C_14_H_10_ 178 Da 0.714	169‰ ± 49‰	n.d.	−192‰ ± 4‰
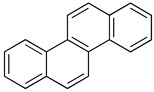 Chrysene	C_18_H_12_ 228 Da 0.667	−174‰ ± 166‰	−272‰ ± 12‰	−143‰ ± 154‰
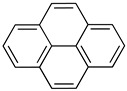 Pyrene	C_16_H_10_ 202 Da 0.625	−10‰ ± 99‰	−233‰ ± 7‰	−238‰ ± 134‰
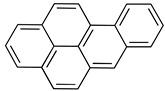 Benzo[a]pyrene	C_20_H_12_ 252 Da 0.6	−12‰ ± 7‰	n.d.	n.d.
Fluoranthene Family: Five-Membered Containing Fully or Partially Aromatic (Nonalternant) PAHs				
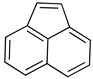 Acenaphthylene	C_12_H_8_ 152 Da 0.667	107‰ ± 7‰	n.d.	n.d.
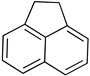 Acenaphthene	C_12_H_10_ 154 Da 0.833	58‰ ± 41‰	n.d.	65‰ ± 47‰
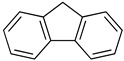 Fluorene	C_13_H_10_ 166 Da 0.769	76‰ ± 14‰	59‰ ± 14‰	−83‰ ± 102‰
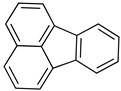 Fluoranthene	C_16_H_10_ 202 Da 0.625	−23‰ ± 67‰	n.d.	−450‰ ± 32‰
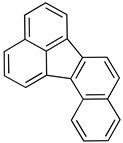 Benzo[j]fluoranthene	C_20_H_12_ 252 Da 0.6	66‰ ± 25‰	n.d.	n.d.

* Error estimates were based on repeated analysis of internal and external standards and included isotopic fractionation associated with the extraction process, solvent reduction steps, and chromatography, as well as instrument effects that can be affected by ionization efficiency, rising baseline, and the generation of H_3_^+^ species.

**Table 3:** Comparison of measurements for individual PAHs and populations of PAHs in the aromatic fraction, insoluble organic matter (IOM), and extraction residues that include both IOM and mineral fraction. Alteration.

Meteorite	Bulk Rock δD	Extraction Residue δD	IOM δD	Aromatic Fraction δD	Fluoranthene δD	Pyrene δD
A-881458 (CM2, very weakly heated)			+903‰ [47]		−219‰ [38] ^d^	−168‰ [38] ^d^
LON 94101 (CM 2; 1.8 [43]/2.6 [44])	−154.6 ± 3.1‰ [48]	−105 ± 0.06‰ ^b^	+1093‰ [49]		−450 ± 32‰ ^b^	−238 ± 134‰ ^b^ + 57‰ [39] ^e^
Murchison (CM 2; 1.6 [43]/2.5 [44])	−74.9 ± 0.9‰ [48]	−42 ± 0.06‰ ^b^	+986‰ [47] +696‰ [49] +777 ± 27‰ [50]	+244‰ [37] ^c^	−560‰ [38] ^d^	−233 ± 7‰ [47] −421‰ [38] ^d^ +357 ± 30‰ [39] ^e^
ALH 83100 (CM ½; 1.1 [43]/2.0 [44])	−201 ± 0.5‰ [41]	−155 ± 0.06‰^b^	+723 ± 11‰ [51]		−23 ± 67‰ ^b^	−10‰ ^b^
Orgueil (CI1; 1.1 [43]/2.1 ^a^)	+138 ± 15.3‰ [48]		+980‰ [49] +972 ± 2‰ [51] +1360 ± 40‰ [36]		+602‰ [52]	+710‰ [52]

^a^ Assuming that the Rubin et al. 2007 [44] scale also applies to CI chondrites. ^b^ This study; ^c^ hot water and methanol extraction residues; ^d^ benzene–methanol extracts; ^e^ hot water extraction residues.

## Data Availability

Supplementary tables provided.

## Supplementary Materials

The following supporting information can be downloaded at: www.mdpi.com/article/10.3390/life12091368/s1.
